# Injuries to the Gum Septum

**Published:** 1892-08

**Authors:** 


ARTICLE III.

### INJURIES TO THE GUM SEPTUM.

CONCLUSION OF AN ARTICLE BY DR. BLACK UPON "INTERPROXIMATE SPACES."

I will now refer more particularly to injuries of the gum septum during operations upon proximate surfaces. This tissue suffers as little, perhaps, from temporary abuse as any other, but it is liable to serious injury from long continued maltreatment. One of the most constant abuses occurs in cases in which the treatment of root canals, or exposed pulps, requires some kind of temporary fillings. In these, it seems to be common practice to fill both the cavity in the tooth and the interproximate space with cotton saturated with some kind of gum or with gutta-percha forced into position without reference to compression of the gum tissue. Indeed severe compression is often recommended as a means of getting the gum tissue out of the way while making the filling. In this way the gum septum is often destroyed, or a deep pocket formed centrally between the teeth. After the treatment is finished and the cavity filled, even when the proximate space and contact are left in good form, this gum septum often fails to recover; or is only partially restored to its former fullness and strength. If the proximate contact is faulty it is irretrievably lost, for the vacant interproximate space or the pocket which has been formed centrally between the teeth, fills with debris which undergoes decomposition with the formation of acid in contact with the unprotected margins of the newly placed filling. This insures a recurrence of decay even though a perfect filling has been made.

The destruction of the gum septum may have been regarded as legitimate in times past, especially after we had cohesive gold and before we had rubber dam. The mode of practice has, in a manner, been inherited by us of the present day. But close observation of cases has shown its evil results, while improved methods demonstrated that this mutilation is unnecessary. It is easy to take a thin blade, such as one of Harlan's scalers, or a somewhat broader blade of the same pattern, and place one edge of it against the neck of the tooth operated upon and lean the other against the proximating tooth, and while holding this rigidly in place insert a gutta-percha filling firmly and at the same time protect the gum septum and give it sufficient space. This requires almost no time. It is convenient to have two or three widths of these blades for the purpose. Cotton and sandarac is an abomination and should have no place in a well regulated office.

In wedging teeth, injury to the gum septum is far too common. In times past many of us have driven wooden wedges between the teeth, crushing out the entire gum septum at a blow. I hope that mode of separating teeth is not practiced by any of us now, for it was essentially bad. I only allude to it to show how our profession has grown up in disregard of the contents of the interproximate space.

Although we may no longer drive such wedges, we are often guilty of destroying this tissue in wedging with rubber, cotton, etc., by injudiciously placing the wedge so that the gum is compressed, or by allowing it to slip from the proximate contact, where it should always be held, into the wider part of the space against the septum of the alveolar process, where, besides causing the patient extreme pain, it will do a lasting injury to the gum tissue. Possibly but few of us are entirely free from this, as an accident of practice, but to do this frequently, or to fail to observe great care to avoid it, and to use all means to mitigate the injury in cases of accident.

Injury to the gum septum in finishing proximate fillings often becomes a serious matter. Think of pushing and pulling a separate file back and forth to file down a proximate filling with the saw on its edge, tearing the soft tissue at every stroke, often until the last of the gum septum is lacerated beyond recovery, if not entirely removed from the space. Then the fillings are finished with flat proximating surface that will catch and hold food between them and prevent the recovery of any gum tissue that may be left in the space.

The separating file should not be used to trim proximate fillings. When it becomes necessary to cut to make room between a newly made proximate filling and the neighboring tooth for passing finishing instruments a thin separating file might be used, but a fine saw held in rigid frame is better. This should be used carefully to make the one cut and then immediately laid aside. Every instrument used afterward should present a smooth polished edge to the gum tissue. The instrument with which I do the bulk of the trimming is the thread saw. This is turned with its smooth polished back to the gum tissue and cuts toward the occluding surface of the tooth. It is insinuated under the overhanging portions of the filling, and the trimming is expeditiously done with the minimum of injury to the gum. The trimming about the buccal, labial, or lingual angles, that this instrument will not reach, is done by the smooth edged draw files and with disks, and the contact point is rounded to the desired form. This work cannot be done with the same expedition and effectiveness with the separating file. Its shape is unsuited to the work.

Many dentists are using the revolving disk for finishing proximate surfaces. So far as the form of the proximate contact is concerned this is as bad as the separating file. The tendency of the disk is to cut away the prominence of the contact point, and this occurs almost inevitably when the endeavor is made to finish the proximate surface with it. Yet the disk is a very useful instrument for rounding the buccal or lingual angles of proximate fillings. I make much use of it for this purpose, but do not use it between the teeth, except occasionally a rim disk when I have plenty of room to pass the rim safely beyond the contact point.

Now it may be said that the dental profession have used flat separating files for forming the proximate surfaces of fillings from time immemorial, and certainly their fillings have not all failed. Certainly not. Many good fillings have been made in spite of the unsuitableness of the flat files, and there has been a continuous advance in the treatment of proximate surfaces up to the present time. So please do not consider me pessimistic in this matter. But many teeth of bad form and faulty texture have never decayed. Many contact points, flattened by wear, have never leaked food into the proximate space. Also, many fillings of bad form have done excellent service. Yet it is the teeth of bad form and faulty texture that decay most. It is the badly worn contacts that oftenest do damage by leakage. It is the fillings of bad form that are most liable to failure.—*Dental Review.*

